# Supplementary material for: Melatonin Delays Postharvest Senescence through Suppressing the Inhibition of BrERF2/BrERF109 on Flavonoid Biosynthesis in Flowering Chinese Cabbage
Source: Int J Mol Sci. 2023 Feb 2;24(3):2933. doi: 10.3390/ijms24032933 (PMC9918124; doi:10.3390/ijms24032933)
Supplement: Supplementary file 1 [file ijms-24-02933-s001.zip › ijms-2175883-supplementary.pdf]

Melatonin delays postharvest senescence through suppressing the inhibition of BrERF2/BrERF109 on flavonoid biosynthesis in flowering Chinese cabbage

Lingqi Yue[1], Yunyan Kang[1], Min Zhong[1], Dengjin Kang[1], Puyan Zhao[1], Xirong Chai[1], Xian Yang [1]*

[1]College of Horticulture, South China Agricultural University, Guangzhou 510642, China

*** Correspondence:**

Corresponding Authors

Xian Yang (yangxian@scau.edu.cn)

+86-135-0305-1303 (X. Yang)

Fax: +86-020-85280228

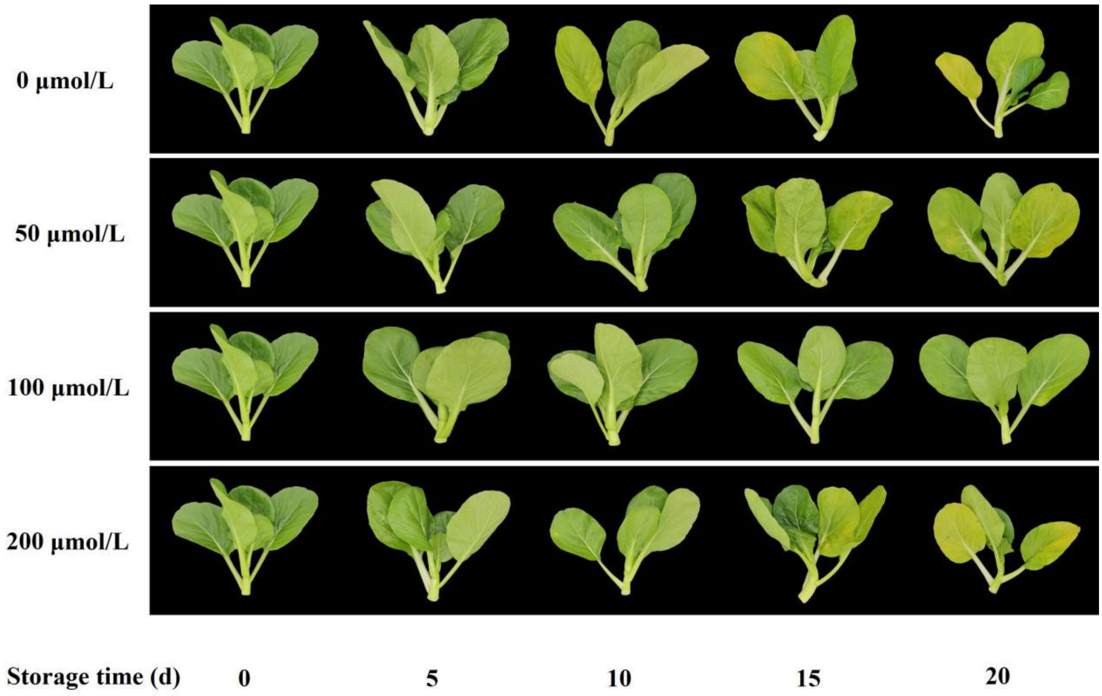

Figure S1. Phenotypic diagrams treated with 0 μmol/L, 50 μmol/L, 100 μmol/L and 200 μmol/L melatonin during storage.

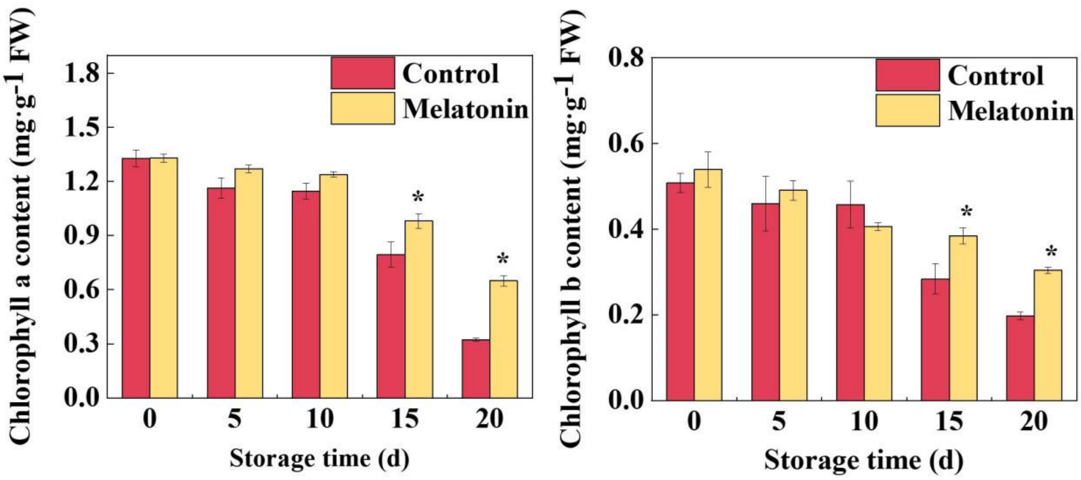

Figure S2. Changes of chlorophyll a and chlorophyll b contents during storage of flowering Chinese cabbage between melatonin-treated and control. Vertical bars represent the standard errors of the means of triplicate assays. Asterisks indicate the means that are statistically significant differences between melatonin-treated and control leaves. (Duncan's multiple range test, *$P < 0.05$).

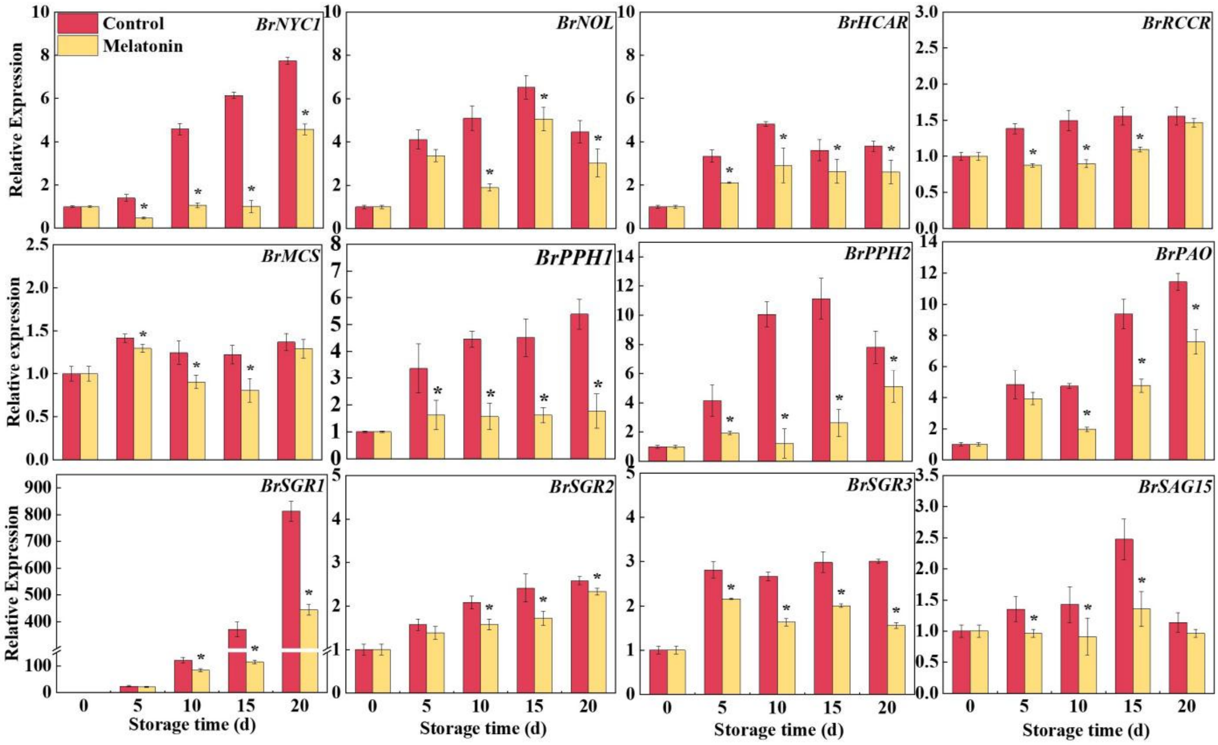

Figure S3. The expression levels of chlorophyll degradation genes and senescence genes in flowering Chinese cabbage leaves during storage between melatonin-treated and control. Data are the mean ± SD of three biological replicates. Asterisks indicate data statistically significant in melatonin-treated leaves compared with control leaves (Duncan's multiple range test: $*P < 0.05$)

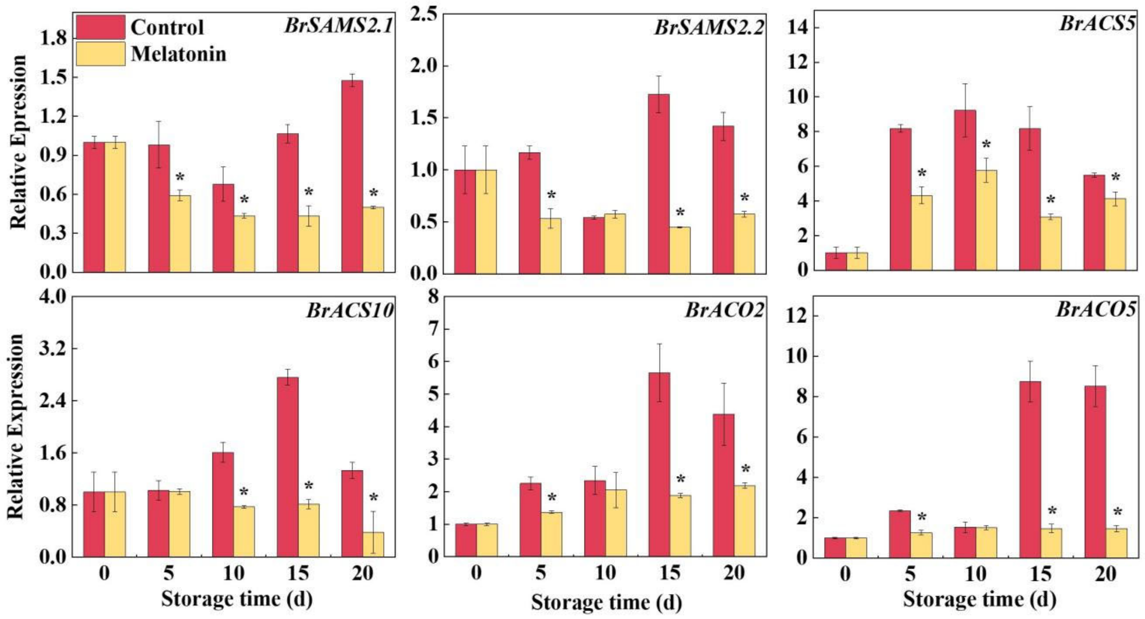

Figure S4. The expression levels of ethylene synthesis genes in flowering Chinese cabbage leaves during storage between melatonin-treated and control. Data are the mean ± SD of three biological replicates. Asterisks indicate data statistically significant in melatonin-treated leaves compared with control leaves (Duncan's multiple range test: *$P < 0.05$)

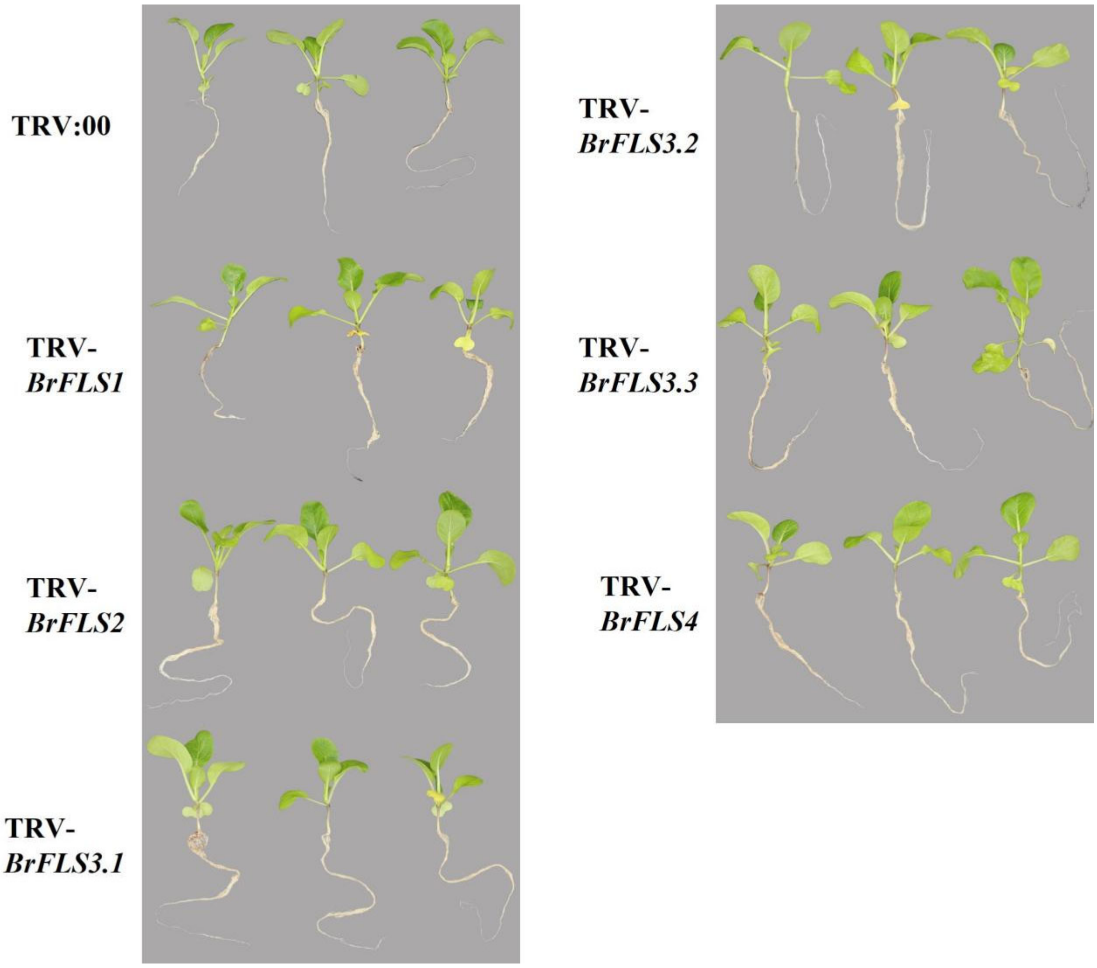

Figure S5. The plant phenotypes of TRV2 and TRV-*BrFLSs* 20 days after silencing.

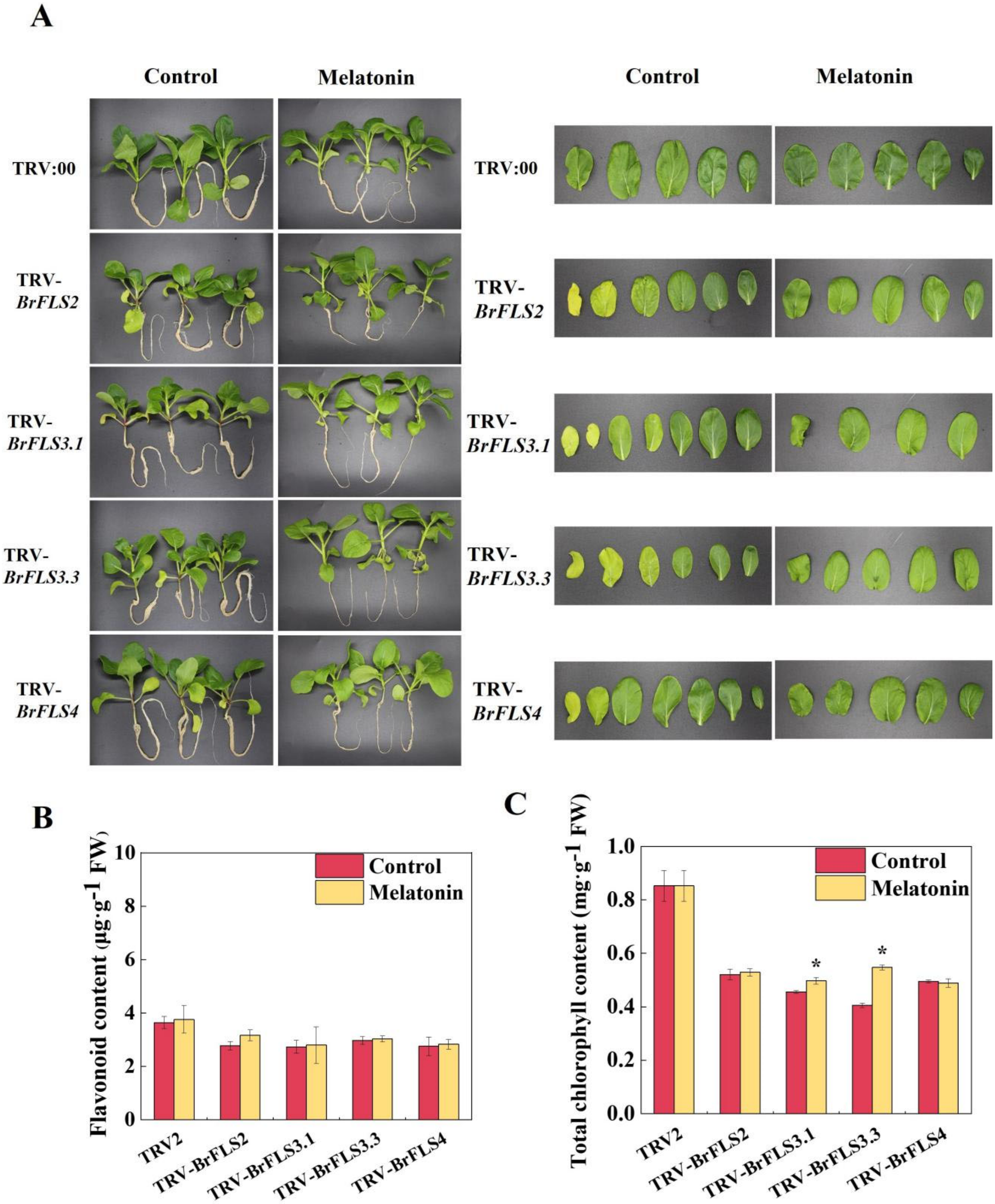

Figure S6. A, The plant and leaf phenotypes of TRV2 and TRV-*BrFLSs* 30 days after silencing. B-C, Flavonoid content (B) and Chlorophyll content (C) in leaves of TRV2, and TRV-*BrFLSs* plants under control and melatonin treatment. Values represent the means ± SD from three repeats. Asterisks indicate the means that are statistically significant differences between melatonin-treated and control leaves. (Duncan's multiple range test, $*P < 0.05$).

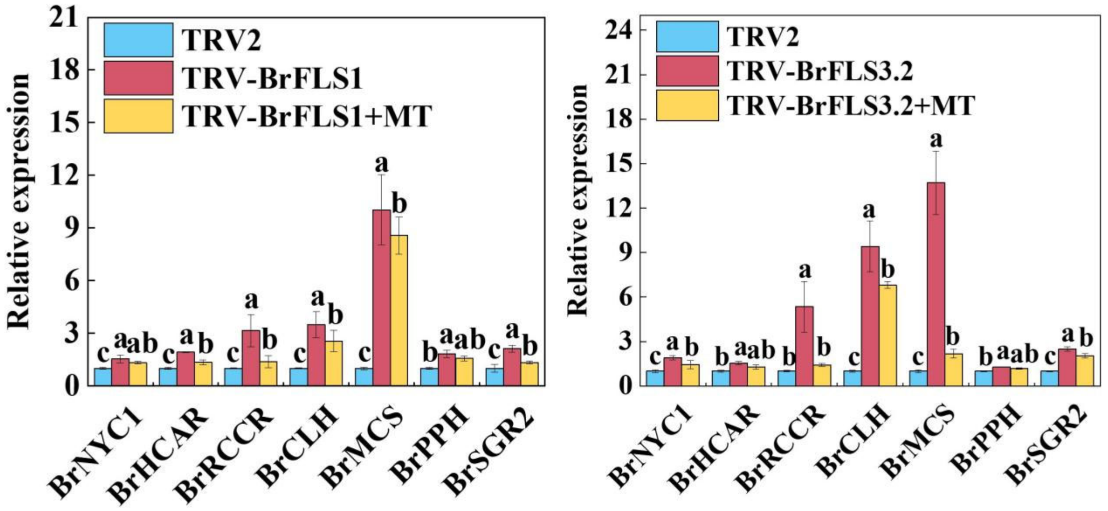

Figure S7. The expression levels of chlorophyll degradation genes and senescence genes in TRV2, TRV-*BrFLS1* and TRV-*BrFLS3.2* plants (30 days after silencing). MT: melatonin treatment. Values represent the means ± SD from three repeats. Letters indicate the significant differences among TRV2, TRV-*BrFLSs* and TRV-*BrFLSs*+MT. (Duncan's multiple range test: *$P < 0.05$)

Query	Hit type	PSSM-ID	From	To	E-Value	Bitscore	Accession	Short name	Incomplete	Superfamily
Q#1 - >BrERF2-like1	specific	197689	118	182	7.61571e-30	105.425	smart00380	AP2	-	cl00033
Q#2 - >BrERF2-like2	specific	197689	122	186	4.24005e-33	113.899	smart00380	AP2	-	cl00033
Q#3 - >BrERF2	specific	197689	137	195	3.26357e-32	112.358	smart00380	AP2	-	cl00033
Q#4 - >BrERF3-like1	specific	197689	26	89	5.5222e-33	113.514	smart00380	AP2	-	cl00033
Q#5 - >BrERF3-like2	specific	197689	22	85	6.04685e-21	81.9277	smart00380	AP2	-	cl00033
Q#6 - >BrERF3-like3	specific	197689	22	85	2.34922e-22	86.1649	smart00380	AP2	-	cl00033
Q#7 - >BrERF3-like4	specific	197689	22	85	6.85002e-20	79.6165	smart00380	AP2	-	cl00033
Q#8 - >BrERF109-like1	specific	197689	137	199	1.51345e-23	90.4021	smart00380	AP2	-	cl00033
Q#9 - >BrERF109	specific	197689	140	202	1.2889e-22	88.0909	smart00380	AP2	-	cl00033

Figure S8. Multiple alignment of nine BrERFs transcription factors (BrERF2-like1, BrERF2-like2, BrERF2, BrERF3-like1, BrERF3-like2, BrERF3-like3, BrERF3-like4, BrERF109-like and BrERF109) in the NCBI website.

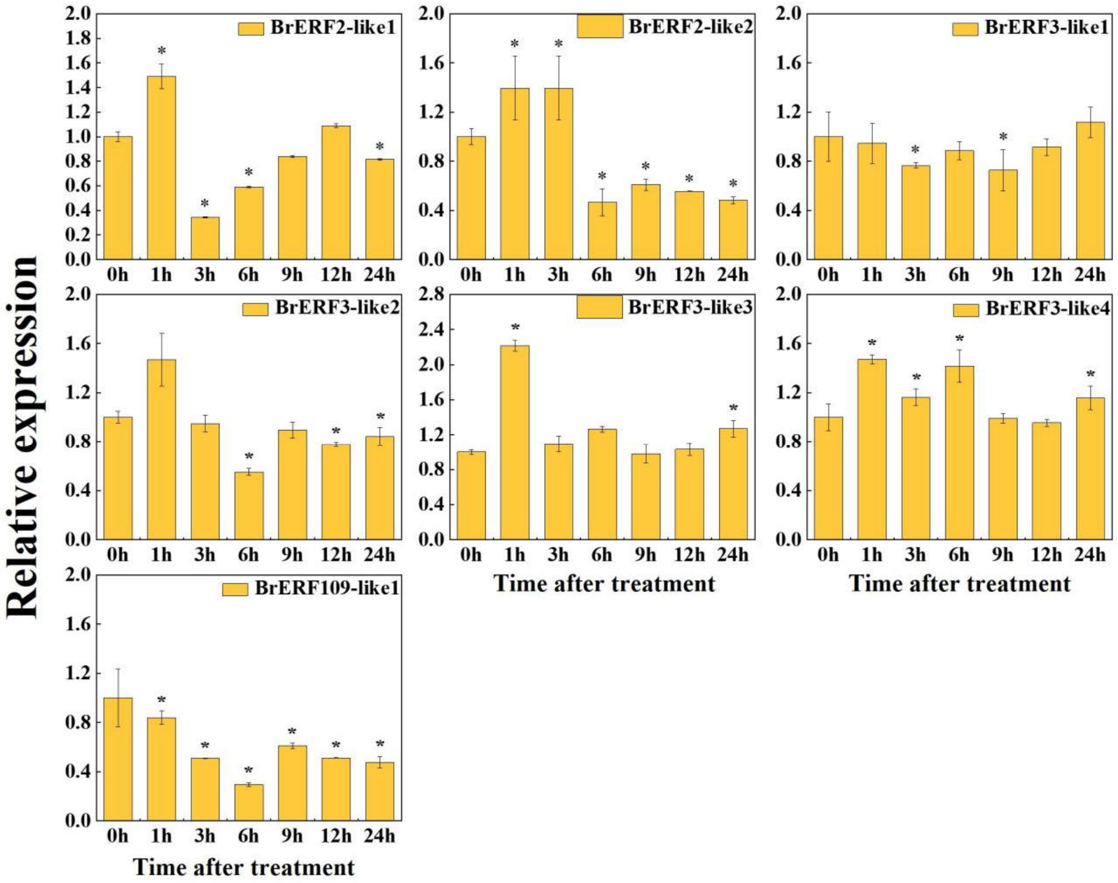

Figure S9. The transcription levels of BrERFs in response to treatment with 100μM melatonin. Values represent the means ± SD from three repeats. Asterisks indicate significant differences (Duncan's multiple range test: *$P < 0.05$).

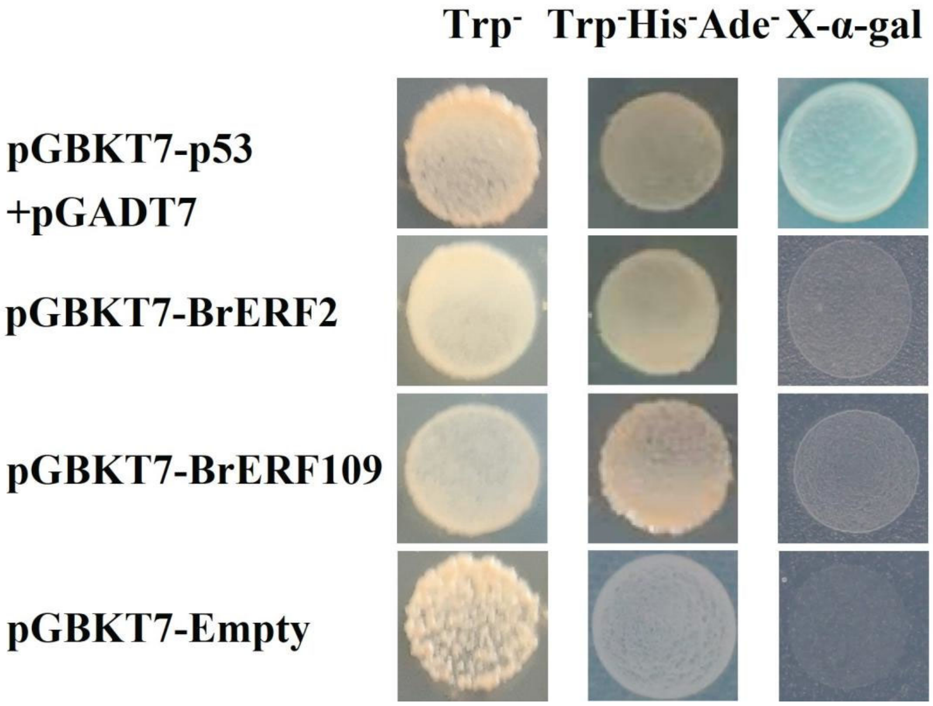

Figure S10. Trans-activation of BrERF2, and BrERF109 in yeast cells. pGBKT7-P53 and pGBKT7 were used as the positive and negative controls, respectively.

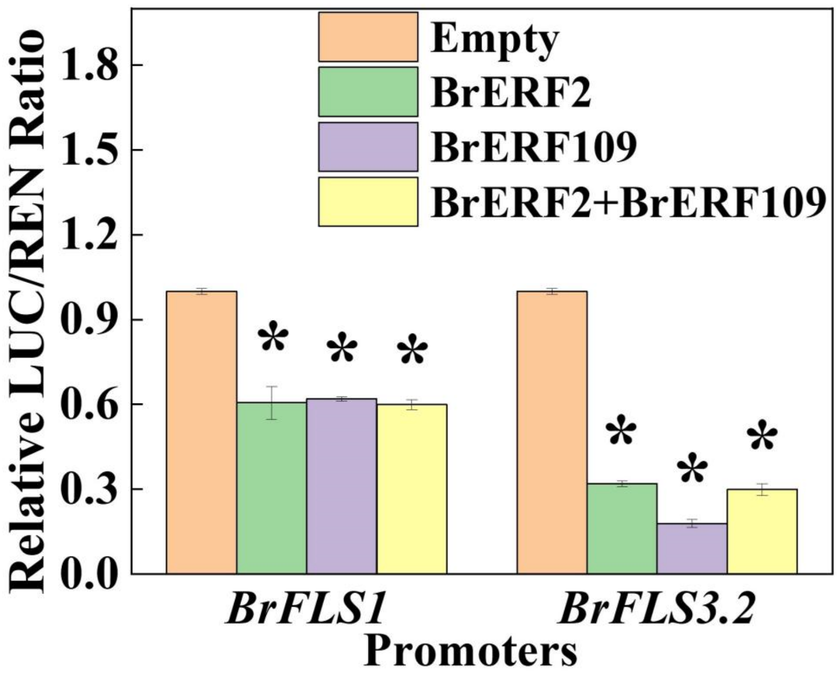

Figure S11. Relative LUC/REN ratio indicated that the transcription levels of flavonoid synthesis genes BrFLS1 and BrFLS3.2 were suppressed by BrERF2, BrERF109, and BrERF2+BrERF109. The independent transfection experiment was repeated three times. Values represent the means ± SD from six repeats. Asterisk indicates significantly different values (Duncan's multiple range test: **P* < 0.05).

Table S1. List of primers used in this study.

Assay	Genes	Primers	Sequences (5' -3')
	BrActin	Actin-F	CGCTTAACCCGAAAGCTAAC
		Actin-R	TACGCCCACTAGCGTAAAG
	BrNYC	NYC1-F	TAGAGAAGGGAAGGATGTGAAGG
		NYC1-R	AAGATATGACCACCCCGAGATT
	BrNOL	NOL-F	TGGTTGGTATTGCCTCTGATG
		NOL-R	TGTCCACCGTTGTCCTGTCT
	BrHCAR	HCAR-F	GTTGAGGCTGTTGTTTGTGTGC
		HCAR-R	GCCCTTCCCTTGTTCCATTG
	BrRCCR	RCCR-F	TCTCAACATAACAAGCATCTCTGG
		RCCR-R	GGTGTCTTGGTAAAAGGTCTGG
	BrMCS	MCS-F	ACTCCTATTCAGAAACCAACCCT
		MCS-R	AACAGTCACTGTGACGATACCGT
	BrPPH1	PPH1-F	TCACAGAACCTCCTGCCTCA
		PPH1-R	GCTAAACTCCCCAAGTCCTCATT
	BrPPH2	PPH2-F	GTATGGGCGGTTGATTTTGTT
		PPH2-R	CGAGATGAGGATGGGTTGCT
	BrPAO	PAO-F	TGGATGAGAACGGACACTTGC
		PAO-R	CCTAGGGGGTTGAACGGAAT
	BrSGR1	SGR1-F	GTCTTTCGGATTTTGCGGAT
		SGR1-R	CCAGCATGGCTCAAGTCTTGT
qRT-PCR	*BrSGR2.1*	SGR2.1-F	CAAGAATCTCCTGTTTGGGTTTA
		SGR2.1-R	AGAGTTTCACATTTTTTGCGTGT
	BrSGR2.2	SGR2.2-F	GCACTAACTAACGACGCCAATG
		SGR2.2-R	CCTTTCCCTTACCTATCCCAAT
	BrSAG15	SAG15-F	CTTCTAAAGACGCTTCCCCTAAT
		SAG15-R	CCGCAATACTAATCGCTAGTCC
	BrPAL3	PAL3-F	TTCCGCCATGTTTGTTGAGG
		PAL3-R	TATGTTCCATGATTGCCGCG
	BrC4H	C4H-F	TGACTTCAGGTATGTGCCGT
		C4H-R	CTGGAGGAGGAAGTAGCTCG
	Br4CL	4CL-F	ATGCTGTGTGGGCTTAGAGT
		4CL-R	CTGACGGCATCTTCAAGCTC
	BrFLS1	FLS1-F	ACACCGAAGCCATCCCTCTC
		FLS1-R	CGTGATTAACCACCTGGAAAAT
	BrFLS2	FLS2-F	GGAAGTCAAGAAAGACCAGCAC
		FLS2-R	TCCGTATCAGATCCATCGAGAT
	BrFLS3.1	FLS3.1-F	CGTGAAAGCGAGCGAAGAAT
		FLS3.1-R	CCAAATGCGGTGAAAGAGATG
	BrFLS3.2	FLS3.2-F	CCAGCGTCAAGAATCAACTACA
		FLS3.2-R	GGACACGGCGGATAGAAAAT
	BrFLS3.3	FLS3.3-F	ATCTTCTATCCGCCGTGTCC

		FLS3.3-R	TTGCTCATCCTCTTGATTTGGT
	BrFLS4	FLS4-F	CCAAGAGTGGGGGGTTTTTC
		FLS4-R	CCTGCCTGTATTCTGGAGGATTT
	BrSAMS2.1	SAMS2.1-F	GTCAACATCGAGCAGCAGAG
		SAMS2.1-R	CTTGGTGGCAAGGACATGAC
	BrSAMS2.2	SAMS2.2-F	CTTACATCGTGAGGCAAGCC
		SAMS2.2-R	CCCATTGCCTCCTCTCTTCA
	BrACS5	ACS5-F	AGAAACGGCTCAAGTCCAGA
		ACS5-R	AACAAACCCGCATTGCTTCT
	BrACS10	ACS10-F	CGGTTCAGCTCACAACACAA
		ACS10-R	CCACAGCCGTTTTGAGTTCA
	BrACO2	ACO2-F	ACCGAGTACCCGAGTTTTGT
		ACO2-R	AAGTCTCTACGGCTGCTGTT
	BrACO5	ACO5-F	TGAGAACTTGGGCTTGCCTA
		ACO5-R	ACAAGGTGGGTAATGGCTGA
	BrERF2-like1	ERF2-like1-F	CGGGGAAGGGAAAGCATTAC
		ERF2-like1-R	GCAGAAGAGGACGACGAAGAAG
	BrERF2-like2	ERF2-like2-F	AGCATTACAGAGGGGTGAGACA
		ERF2-like2-R	AACCACGCATCCTAAAAGCAG
	BrERF2	ERF2-F	ATTATGGCGGCAACAGAAACC
		ERF2-R	AAATCAAAAAAATCGCTGAGACA
	BrERF3-like1	ERF3-like1-F	CTTCCCCTCTCCAACCACTC
		ERF3-like1-R	ATGTCGTCTCCATCATCAATCAC
	BrERF3-like2	ERF3-like2-F	TTCAGAGGCGTGAGGAAGAGA
		ERF3-like2-R	GGGAGGAGAAGGAGGGGACT
	BrERF3-like3	ERF3-like3-F	TCTCGCTCCGAATCAGAACC
		ERF3-like3-R	CAGTCGCTGTGGCAATCCTC
	BrERF3-like4	ERF3-like4-F	CGTGACCCCTTGAAAAAATCC
		ERF3-like4-R	GCTGATGATAACAACCTCCTGCT
	BrERF109-like1	ERF109-like1-F	GGAAGGAGGAGGCAAAGTGA
		ERF109-like1-R	AGACGAAGTGTAATCCGCAAAC
	BrERF109	ERF109-F	GACGCCGTATCAGGGCTTCT
		ERF109-R	CGTGCTCTCTCTTCCACTACTCTT
Virus-induced gene silencing	*BrFLS1*	FLS1-VIGS-F	GTGAGTAAGGTTACCGAATTCTGAAGAATGGGGGATTTTCC
		FLS1-VIGS-R	CGTGAGCTCGGTACCGGATCCAACGCGTACTCTTCGTTCACC
	BrFLS2	FLS2-VIGS-F	GTGAGTAAGGTTACCGAATTCCAAGAGTGGGGGATATTTCAC
		FLS2-VIGS-R	CGTGAGCTCGGTACCGGATCCGGTCTCCTTTGTGTACTCTTCAG
	BrFLS3.1	FLS3.1-VIGS-F	GTGAGTAAGGTTACCGAATTCGCCGGAGCTGATACGGCGGT
		FLS3.1-VIGS-R	CGTGAGCTCGGTACCGGATCCGATAGCCATTCCATG

			ATTTTCTCCGAT
	BrFLS3.2	FLS3.2-VIGS-F	GTGAGTAAGGTTACCGAATTCTGGACTTCAGGCGTACCAGG
		FLS3.2-VIGS-R	CGTGAGCTCGGTACCGGATCCCAGTCGATAGGAAGCTTGTTCATC
	BrFLS3.3	FLS3.3-VIGS-F	GTGAGTAAGGTTACCGAATTCCGGACTTCAGGCGTACCAGG
		FLS3.3-VIGS-R	CGTGAGCTCGGTACCGGATCCATTTCGAGAGGAAGCTTGTTCATCT
	BrFLS4	FLS4-VIGS-F	GTGAGTAAGGTTACCGAATTCACAGGCGAAGAAGCTCGCAG
		FLS4-VIGS-R	CGTGAGCTCGGTACCGGATCCCCGATAAGAATATTGATGATAGATGGG
	BrERF2	ERF2-VIGS-F	GTGAGTAAGGTTACCGAATTCATGCATTATCCTAATAACACCAG
		ERF2-VIGS-R	CGTGAGCTCGGTACCGGATCCCTTTTCGATTCTCTGATTTGG
	BrERF109	ERF109-VIGS-F	GTGAGTAAGGTTACCGAATTCACGGAGTTACAATTCAGTGAGTCAAT
		ERF109-VIGS-R	CGTGAGCTCGGTACCGGATCCACCTCCTTCGGCTTCTCC
Subcellular localization analysis	*BrERF2*	ERF2-1300-F	GAGCTCGGTACCCGGGGATCCATGTATGGAGAGGGCGAGATT
		ERF2-1300-R	GCCCTTGCTCACCATGTCGACTGCAACTTCGCACTTCACCT
	BrERF109	ERF109-1300-F	GAGCTCGGTACCCGGGGATCCATGCATTATCCTAATAACACCAG
		ERF109-1300-R	GCCCTTGCTCACCATGTCGACCTGAAACATATCAGCAATAGTGTT
Transcriptional assay	*BrERF2*	ERF2-BD-F	ATGGCCATGGAGGCCGAATTCATGTATGGAGAGGGCGAGAT
		ERF2-BD-R	TAGTTATGCGGCCGCTGCAGTTATGCAACTTCGCACTTCAC
	BrERF109	ERF109-BD-F	ATGGCCATGGAGGCCGAATTCATGCATTATCCTAATAACACCAG
		ERF109-BD-R	TAGTTATGCGGCCGCTGCAGTCACTGAAACATATCAGCAATAGT
Dual-LUC assay	*BrERF2*	ERF2-62SK-F	CGGGCTGCAGGAATTCATGTATGGAGAGGGC
		ERF2-62SK-R	CGGTATCGATAAGCTTTTATGCAACTTCGCA
	BrERF109	ERF109-62SK-F	CGGGCTGCAGGAATTCATGCATTATCCTAAT
		ERF109-62SK-R	CGGTATCGATAAGCTTTCACTGAAACATATC
	BrFLS1	FLS1-0800-F	TATAGGGCGAATTGGGTACCATCGCAATCTCAATCGAATTTT
		FLS1-0800-R	TATGTTTTTGGCGTCTTCCATGGGCTGCTTTGTTATGC

			TTCGTT
	BrFLS3.2	FLS3.2-0800-F	TATAGGGCGAATTGGGTACCACCACCTCTGTAAGCTCCTCTGT
		FLS3.2-0800-R	TATGTTTTTGGCGTCTTCCATGGGTGGCATGAGAAGGCAAAC
	BrERF2	ERF2-AD-F	GCCATGGAGGCCAGTGAATTCATGTATGGAGAGGGCGAGAT
		ERF2-AD-R	CAGCTCGAGCTCGATGGATCCTTATGCAACTTCGCACTTCAC
	BrERF109	ERF109-AD-F	GCCATGGAGGCCAGTGAATTCATGCATTATCCTAATAACACCAG
		ERF109-AD-R	CAGCTCGAGCTCGATGGATCCTCACTGAAACATATCAGCAATAGT
Y1H assay	*BrFLS1*	FLS1-AbAi-F	GAAAAGCTTGAATTCGAGCTCATCGCAATCTCAATCGAATTTT
		FLS1-AbAi-R	ATACAGAGCACATGCCTCGAGGCTGCTTTGTTATGCTTCGTT
	BrFLS3.2	FLS3.2-AbAi-F	GAAAAGCTTGAATTCGAGCTCACCACCTCTGTAAGCTCCTCTGT
		FLS3.2-AbAi-R	ATACAGAGCACATGCCTCGAGGTGGGCATGAGAAGGCAAAC
	BrERF2	ERF2-RI101-F	TCTTCACTGTTGATACATATGATGTATGGAGAGGGCGAGAT
		ERF2-RI101-R	AGAGTTGTTGATTCAGAATTCTTATGCAACTTCGCACTTCAC
	BrERF109	ERF109-RI101-F	TCTTCACTGTTGATACATATGATGCATTATCCTAATAACACCAG
GUS analysis		ERF109-RI101-R	AGAGTTGTTGATTCAGAATTCTCACTGAAACATATCAGCAATAGT
	BrFLS1	FLS1-BI101-F	CTTGCATGCCTGCAGGTCGACATCGCAATCTCAATCGAATTTT
		FLS1-BI101-R	AAGGGACTGACCACCCGCTGCTTTGTTATGCTTCGTT
	BrFLS3.2	FLS3.2-BI101-F	CTTGCATGCCTGCAGGTCGACACCACCTCTGTAAGCTCCTCTGT
		FLS3.2-BI101-R	AAGGGACTGACCACCCGTGGGCATGAGAAGGCAAAC
	BrERF2	ERF2-nYFP-F	CTGAGGAGGATCTTGGATCCATGTATGGAGAGGGCGAGAT
		ERF2-nYFP-R	CCGAATTCACTAGTGTCGACTTATGCAACTTCGCACTTCAC
BiF	*BrERF109*	ERF109-cYFP-F	TTCCAGATTACGCTGGATCCATGCATTATCCTAATAACACCAG
		ERF109-cYFP-R	CCGAATTCACTAGTGTCGACTCACTGAAACATATCAGCAATAGT
